# Lignin: Characterization of a Multifaceted Crop Component

**DOI:** 10.1155/2013/436517

**Published:** 2013-11-14

**Authors:** Michael Frei

**Affiliations:** Division of Abiotic Stress Tolerance in Crops, Institute of Crop Science and Resource Conservation (INRES), University of Bonn, Karlrobert-Kreiten Straße 13, 53115 Bonn, Germany

## Abstract

Lignin is a plant component with important implications for various agricultural disciplines. It confers rigidity to cell walls, and is therefore associated with tolerance to abiotic and biotic stresses and the mechanical stability of plants. In animal nutrition, lignin is considered an antinutritive component of forages as it cannot be readily fermented by rumen microbes. In terms of energy yield from biomass, the role of lignin depends on the conversion process. It contains more gross energy than other cell wall components and therefore confers enhanced heat value in thermochemical processes such as direct combustion. Conversely, it negatively affects biological energy conversion processes such as bioethanol or biogas production, as it inhibits microbial fermentation of the cell wall. Lignin from crop residues plays an important role in the soil organic carbon cycling, as it constitutes a recalcitrant carbon pool affecting nutrient mineralization and carbon sequestration. Due to the significance of lignin in several agricultural disciplines, the modification of lignin content and composition by breeding is becoming increasingly important. Both mapping of quantitative trait loci and transgenic approaches have been adopted to modify lignin in crops. However, breeding goals must be defined considering the conflicting role of lignin in different agricultural disciplines.

## 1. Introduction

Lignin is a complex aromatic polymer, which is deposited in the secondary cell walls of all vascular plants [1, 2]. It is tightly cross-linked with other cell wall components and can thus be considered the “cellular glue” providing strength to plant tissues and fibers and stiffness to the cell walls [3]. Its function in plants also includes the defense against abiotic and biotic stresses, especially pathogens and insects [4], and conferring stability to xylem vessels for efficient water transport [5]. Together with the carbohydrate polymers cellulose and hemicellulose, lignin forms the largest portion of “lignocellulosic” plant materials. Thus, lignin accounts for a substantial portion of the total organic carbon in the biosphere, surpassed only by cellulose [2, 6]. 

It has been estimated that more than 2 × 10^11^ tons of lignocellulosic material are produced as agricultural byproducts each year, including straw, roots, husks, bagasse, shells [7]. Cereal production alone produces roughly 2.8 × 10^9^ tons of lignocellulosic crop residue each year [8]. A large portion of these crop residues are traditionally incorporated into soils, but some are used as animal feed; lignocelluloses have recently been identified as an abundant source of feedstock for bioenergy production [7, 9]. With the increasing demand for biomass as feedstock for bioenergy conversion, even the production of specialized biomass crops such as *Miscanthus* spp. or bioenergy maize is becoming more common, thus adding to the vast pool of lignocellulosic material produced each year [9]. The characteristic feature determining the role of lignin in various applications of lignocelluloses is its resistance to microbial fermentation, whether in living plants, in ruminants' digestive tract, in soils, or in bioenergy reactors. 

This review characterizes the role of lignin from the point of view of analytical chemistry, plant stress physiology, animal nutrition, bioenergy production, soil science, and crop breeding. It focuses on annual crops grown in agro-ecosystems, rather than woody and perennial species and natural ecosystems. By elucidating the role of lignin from the perspective of several disciplines, synergies and conflicts are identified that need to be addressed in crop management, the utilization of crop products and residues, and in plant breeding schemes. 

## 2. Lignin Biosynthesis

**Figure 1:**
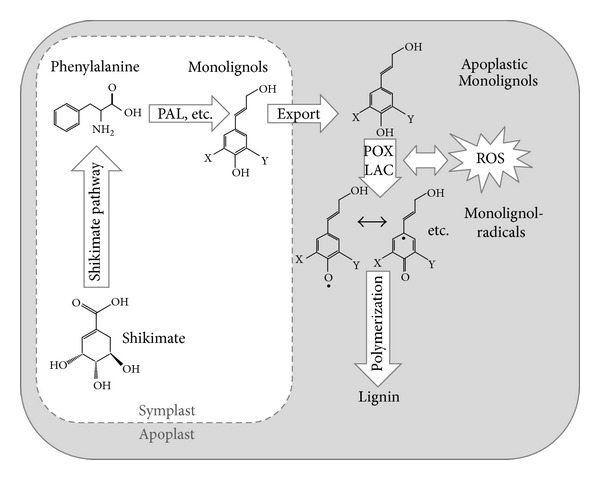
Simplified model of lignin biosynthesis in vascular plants. PAL: phenylalanine ammonia lyase, POX: peroxidases, LAC: laccases, and ROS: reactive oxygen species.

Lignin biosynthesis in plants can be divided into three major phases: (i) synthesis of monolignols in the symplastic shikimate and phenylpropanoid pathway, (ii) export of monolignols to the apoplast, and (iii) activation of monolignols by enzyme-mediated formation of monolignol radicals in the apoplast and their polymerization to form complex lignin polymers (Figure 1). 

The phenylpropanoid pathway is the source of a huge array of secondary metabolites such as flavonoids, anthocyanins, tannins, coumarin, and volatiles [10, 11]. It is based on just a few intermediates of the shikimate pathway, which involves the conversion of the carboxylic acid shikimate to the aromatic amino acid L-phenylalanine. Important steps of the shikimate pathway are localized in the plastids [10]. The deamination of L-phenylalanine to *trans*-cinnamate is catalyzed by the key enzyme phenylalanine ammonia lyase (PAL), which forms the entry point into the synthesis of all phenylpropanoids [11]. The formation of monolignols further requires hydroxylation of the aromatic ring, methylation of hydroxyl groups, and the stepwise reduction of monolignol side chains from carboxylic acids to alcohols. These steps are mediated by specific enzymes [2] and lead to the formation of three major monolignols, that is, *p*-Coumaryl alcohol, Coniferyl alcohol, and Sinapyl alcohol. 

These monolignols constitute the building blocks for lignin polymers and have to be transported across the plasma membrane to the apoplast. Although the transport of monolignols remains poorly understood, three major models have been proposed [11]: (i) transport of monolignols through vesicles derived from Golgi bodies, (ii) passive diffusion of monolignols through the plasma membrane, and (iii) active transport mediated by plasma-membrane located transporters. Further details of these proposed transport mechanism are discussed in a review by Liu [11]. Overall, monolignol transport remains a poorly understood step in lignin biosynthesis that warrants further research. 

Activation of monolignols in the apoplast requires enzymes such as peroxidases (POX), laccases (LAC), or other polyphenol oxidases that transfer electrons from monolignols to electron receptors. These apoplastic enzymes interact with reactive oxygen species (ROS) such as hydrogen peroxide or superoxide, which act as electron receptors or modulators of POX and LAC enzymes through their signaling function [12–14]. ROS are formed as byproducts of many metabolic processes in plants, but can also be actively produced through enzymes such as NADPH-oxidases or “class III peroxidases” (also termed as guaiacol POX), and they accumulate excessively when plants encounter abiotic or biotic stress [14, 15]. POX use hydrogen peroxide as an electron receptor to oxidize a variety of phenolic compounds including monolignols [11]. Laccases (LAC) are copper-containing apoplastic enzymes that oxidize phenolic compounds using molecular oxygen as an electron receptor [11]. Generally, plant genomes contain many isoforms of POX and LAC genes with presumably overlapping functions, making it difficult to link particular isoforms of these enzymes to lignin synthesis [2, 16]. After the activation of monolignols by these enzymes, oxidized monolignol radicals couple on to each other to form three dimensionally cross-linked structures. This process is called polymerization and constitutes the final step of lignin biosynthesis. 

This section gives only a brief summary of the most important processes involved in lignin biosynthesis. The genetic, transcriptional, and biochemical regulation of lignin biosynthesis in plants is extremely complex and has been discussed extensively (for reviews see [2, 6, 11, 16, 17]). 

## 3. Lignin Content of Crops and Quantitative Measurements

The lignin content of crops depends on multiple factors, such as the growth stage, genotype, morphological fraction, and environmental conditions. Data from studies that surveyed a broad range of herbaceous agricultural crops show that lignin concentration in the vegetative tissue usually ranges from 1 to 15 percent of the dry mass [18–26]. The extent of lignification tends to increase with increasing plant age [27–30], an effect that appears to be more pronounced in grasses as compared to legumes [22]. In addition, large genotypic differences in lignin content within the same species have been reported. For example, studies on genotypic variation in rice straw lignin reported values ranging from 1 to almost 12 percent [20, 30], although at least some of this variation might be explained by the fact that the analyses were conducted in different laboratories, as detailed below. Another study on the biomass crop *Miscanthus* revealed a range of 6 to 14 percent in 244 different accessions [19]. Differences also occur between lignification of different morphological fractions. In maize, roots were shown to contain more lignin than aboveground biomass [31], while in rice, differences between lignification of the stem and leaf were not consistent and depended on the genotype [20]. Moreover, abiotic and biotic stress factors tend to affect the lignification of crops [4, 32–34], as detailed in the respective sections of this review. 

Apart from these factors causing true differences in lignification of crops, substantial variation arises from the analytical method employed in determining lignin concentration. Comparative studies testing different analytical methods reported up to fourfold differences in lignin content of identical samples [18, 35–38]. It is thus evident that lignin values obtained using different methods in different laboratories cannot be directly compared. 

**Figure 2:**
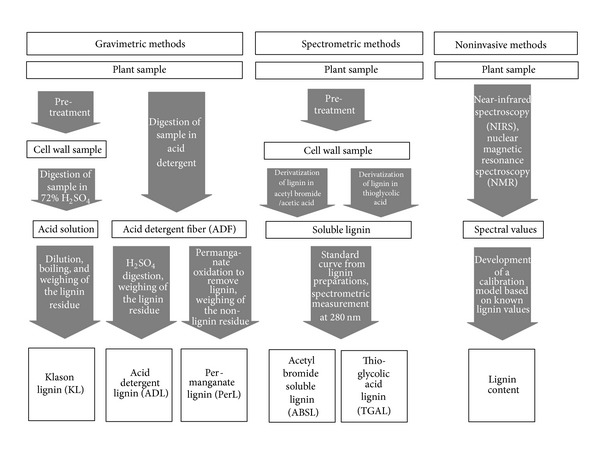
Overview of different lignin analysis methods.

Methods for determining lignin in crops can be grouped into three categories: gravimetric, spectrometric, and noninvasive methods (Figure 2). Several of these methods require a pretreatment of plant samples to remove non-cell wall components such as proteins, lipids, and nonstructural carbohydrates. The type of pretreatment depends on the sample and may consist of a neutral detergent fiber digestion [39], treatment with hot water-organic solvent, or ethanol-benzene [40]. Gravimetric methods are based on sequential digestion and weighing of cell wall fractions. The “Klason” method represents a classical approach [41] and is based on a two-step digestion of all nonlignin components in sulfuric acid, followed by the recovery and weighing of the residue. The acid detergent lignin (ADL) method [39] is based on pretreatment of the samples with an acid detergent solution (ADS), in which proteins, nonstructural carbohydrates, lipids, pectin, and hemicelluloses are removed, leaving a residue of cellulose and lignin. Cellulose is then removed by sulfuric acid digestion, and the residue is weighed. This method may be inaccurate in samples containing high amounts of cutin or suberin, two compounds which are not removed by ADS [35]. In this case, ADS treated samples are exposed to permanganate oxidation, which decomposes the lignin fraction. Permanganate lignin (PerL) is then obtained by the difference in weight before and after permanganate treatment. 

Spectrometric methods are based on solubilization of lignin from cell wall preparations and measurement of its specific absorbance at 280 nm. These methods require the removal of potentially interfering substances by pretreatment as described previously, to obtain cell wall preparations. Lignin is then derivatized using acetylbromide [18, 40], HCl triethylene glycol [40], or thioglycolate [35, 42] to render lignin soluble in a suitable solvent. Solubilized lignin can then be quantified by spectrometric measurements at 280 nm using extinction coefficients, but these need to be calibrated for each type of sample [35]. Alternatively, standard curves for quantification can be obtained using standardized lignin preparations [40, 42, 43]. 

Noninvasive methods take advantage of specific spectra associated with lignin, which can be measured in almost unprocessed samples. However, these methods require calibration using data obtained from gravimetric or spectrometric methods, and the calibration models obtained are specific to a particular type of samples (e.g., rice straw, alfalfa, etc.). Commonly used noninvasive methods include near infrared spectroscopy (NIRS) and nuclear magnetic resonance (NMR) spectroscopy [35, 44]. The advantage of these methods is that, once a calibration model has been established, they are suitable for high throughput analyses in applications such as plant breeding. 

The lowest values are usually obtained from the widely used ADL method, presumable because a portion of the lignin is removed during the acid detergent treatment preceding the sulfuric acid digestion, thus leading to an underestimation of true lignin values [35, 36, 45]. However, the data obtained from different methods strongly depends on the type of sample, its content of substances interfering with each of the assays, and its lignin composition. Further details on comparison of analytical methods, including analytical protocols, have been published elsewhere [18, 35, 39, 40, 42]. 

## 4. Role of Lignin in Abiotic Stress Tolerance

Lignin in crops interacts with abiotic stresses in two ways: (i) many abiotic stresses influence lignin biosynthesis and therefore affect the lignin content of crops [4, 33], and (ii) lignification of crop tissues affects plant fitness and can confer tolerance to abiotic stresses [46, 47]. The effects of some predominant abiotic stresses on lignin content of crops are summarized in Table 1. This summary considers only studies reporting actual lignin measurements and excludes reports on stress responses of lignin biosynthetic genes and enzymes, without actually measuring lignin. Evidently, most of these studies reported enhanced lignin content in crops grown under abiotic stress. 

In the case of drought, contradicting effects on lignin level have been observed (Table 1). Increases in lignin content observed in maize, clover, and ryegrass were explained with drought-induced activation of lignifying enzymes [48, 53, 56]. The role of lignin in the drought tolerance of maize was also confirmed in experiments in which lignin deficient mutants exhibited drought symptoms even in well-watered conditions [150] and in which leaf lignin levels correlated with drought tolerance in a set of contrasting genotypes [56]. Similarly, transgenic tobacco plants with enhanced lignin levels showed improved tolerance to drought compared to the wild type [151]. However, some studies also reported decreases in the lignin content due to drought. For example, it was found that water deficit decreased the level of lignifying enzymes and consequently the lignin level in maize leaves [57]. Another study reported enhanced drought tolerance in transgenic alfalfa plants, which have lower lignin levels than their wild type [152]. The authors explained these observations with constitutive stress defense gene activation associated with lack of lignin in their alfalfa mutants. In summary, controversial results have been reported from drought experiments, which could be explained by species and genotypic differences, different drought treatments, or different methods employed for lignin measurement. 

Unlike drought stress, almost all other experiments reported increases in lignification when crops were subjected to various abiotic stresses (see Table 1), thus highlighting the importance of lignin as a stress response factor. In the case of salinity, lignification of the root was observed in many crop species (Table 1). A transgenic rice line which deposited enhanced levels of lignin in the roots when exposed to salt treatment was more tolerant than its wild type, which did not show such a response [64]. The beneficial effects of lignification were explained with anatomical changes that facilitate water flow and maintain structural integrity of the xylem vessels during salt stress [60, 62], or by lignification of the Casparian strip, which forms a mechanical barrier to ion diffusion in the root endodermis [63, 153, 154]. In fact, lignin was recently shown to be the major component of the Casparian strip *Arabidopsis thaliana* roots [155]. Lignification has also been proposed to be a factor causing root growth reduction under salt stress due to elevated rigidity of lignified cell walls [66].

Similar to salinity, lignification of the root was observed as a response to mineral toxicities in many crop species (Table 1). When chamomile plants were subjected to short-term (7 days) aluminum (Al) toxicity [156], the effects of lignification varied depending on the concurrent application of biochemical regulators: it increased with the application of salicylic acid but decreased with application of the reducing agent dithiothreitol (DTT). In studies on rice and wheat roots, Al toxicity increased the root hydrogen peroxide levels, which was considered as a redox signal leading to lignification [68, 69]. In a study on flax [71], Al toxicity likewise led to lignification of the root, but this effect was mitigated by application of high doses of boron (B) [71], while excess B solely also increased lignin levels. B toxicity also increased lignification of tomato roots [74], while cadmium and copper toxicity stimulated the lignification of soybean roots [72]. Enhanced deposition of lignin in the root endodermis was also observed under Zn toxicity in the metal hyper-accumulator plant *Thlaspi caerulescens*,which can tolerate much higher metal levels than other species [157]. Common observations in all of these studies were that mineral toxicities increased hydrogen peroxide levels, which induced lignin biosynthetic enzymes (especially POX) and consequently led to lignification and solidification of the cell walls but also reduced root growth [158]. 

Mineral deficiencies were also shown to influence the lignin level of crops (Table 1). Nitrogen (N) deficiency led to increases in lignin content of tobacco plants [78], which was explained with a shift from nitrogen containing compounds to carbon-rich phenylpropanoids in the plant tissues. Calcium deficiency induced the activity of enzymes of the phenylpropanoid pathway, which increased the lignin level in soybean roots [79]. Similarly, potassium, phosphorus, and N deficiency increased the lignin content of potato tubers [80], but the authors did not give a physiological explanation for their observation. Manganese (Mn) deficiency appears to be an exception, as it reduced the lignin concentration in wheat roots and shoots [75, 76]. This exception can be explained by the fact that Mn is required for lignin biosynthesis as it activates several enzymes of the shikimate and phenylpropanoid pathway [159, 160]. 

High tropospheric ozone led to increased lignin levels in the aboveground parts of a number of forage or cereal crops (Table 1). Ozone is a phytotoxic air pollutant which enters the crop leaves during photosynthetic gas exchange and decomposes into reactive oxygen species (ROS) in the apoplast [161, 162]. This leads to an oxidative burst, during which further ROS are produced through the function of enzymes such as NADPH oxidase, followed by a signaling cascade which can lead to cell death and the formation of visible leaf symptoms [163]. Genes and enzymes involved in lignin biosynthesis, such as phenylalanine ammonia lyase (PAL) or POX, are triggered by these processes because they form part of the defense mechanism to contain cell death [164, 165]. Thus, there is broad agreement in the scientific literature that ozone exposure leads to enhanced lignin concentration in crops (Table 1). 

Similar mechanisms have been put forward to explain increases in lignin concentration due to elevated UV radiation (Table 1). There is consensus that UV-B or UV-C radiation stimulated the phenylpropanoid pathway leading to enhanced lignification in grasses [88], tomato fruits [89], cucumber seedlings [90], and quinoa seedlings [91], representing a resistance mechanism against oxidative stress. In a study on soybeans [92], high UV-B radiation only increased the level of soluble phenolics but not that of lignin polymers. The authors speculated that the lack of polymerization occurred because samples were taken early in the growing season and emphasized the role of UV-induced phenolics in the protection against insect herbivory as a positive side effect. Similarly, transgenic rice lines with enhanced lignin level showed improved resistance against high UV radiation but also biotic stresses [99].

Apart from its involvement in typical abiotic stresses, lignin has long been assumed to be involved in resistance of crops to lodging, although it remained unclear whether lignin had a positive or negative effect [166]. While lignin could lend mechanical support to the stalks, it may also have the opposite effect of making stalks more brittle and thus susceptible to mechanical damage [166]. This decade-old question is still being discussed in the scientific literature with contradictory results. Some recent work indicated that high lignin levels were associated with lodging resistance in wheat [167, 168] and pea [169], and a quantitative trait locus (QTL) for lodging resistance colocated with a QTL for high lignin content in ryegrass [170]. A study on rice concluded that lignin played an important role in lodging tolerance but suggested that its distribution and density were more important than its concentration [171]. No significant differences in lignin content were seen in wheat cultivars differing in lodging resistance [172], while another study concluded that cellulose rather than lignin conferred resistance to lodging in wheat [173]. In addition, brown-midrib mutations, which were associated with reduced lignin levels, did not affect lodging resistance in maize, sorghum, and pearl millet [174]. A study on a maize mutant with drastically reduced mechanical strength suggested that cellulose rather than lignin deposition in the stalk was associated with susceptibility to lodging [175]. In summary, the question of whether lignin confers tolerance to lodging remains unanswered.

## 5. Role of Lignin in Biotic Stress Tolerance

The cell wall constitutes the first line of defense of plants against pathogens such as bacteria and fungi, nematodes, or herbivorous insects [176, 177]. Lignin solidifies the cell wall, providing a nondegradable barrier for pathogens, and is therefore thought to enhance its protective effect against such biotic stresses [4, 178]. Enhanced lignin biosynthesis due to biotic stress has been ascribed to stimulation of the phenylpropanoid pathway and the induction of apoplastic lignin polymerization [95, 179]. These defense reactions are mediated by ROS-induced signaling cascades. A common response of plants to biotic stresses has been termed “oxidative burst,” and involves the active production of apoplastic ROS [14, 180, 181]. This can be mediated by NAD(P)H-oxidases, that is, plasma-membrane bound enzymes transferring electrons from cytosolic NAD(P)H to extracellular oxygen to produce superoxide. This particularly aggressive ROS is dismutated to hydrogen peroxide by the enzyme superoxide dismutase (SOD) [180]. Alternatively, an oxidative burst can be mediated by apoplastic enzymes such as class III POX [13] or polyamine oxidases [181]. Apoplastic ROS serve as signaling molecules to induce defense reactions and serve as electron receptors for lignification, which facilitates the containment of pathogens and wound-healing. 

These processes have been observed in numerous studies investigating a broad range of biotic stresses affecting model plants, woody plant species, and agricultural crops. Experiments supporting the protective effects of lignification against biotic stresses in agricultural crops are summarized in Table 2. The broad spectrum of different crops and pathogens/insects highlights the general applicability of the principle that lignin constitutes a biotic stress tolerance factor. The majority of the experiments reporting a protective role of lignin dealt with fungal pathogens, suggesting that lignification is particularly effective against this category of pathogens. Fewer studies dealt with bacteria, nematodes, and insects. The experiments summarized in Table 2 can be grouped into three categories.

- The first category includes studies reporting indirect or correlative evidence for the involvement of lignin in tolerance to abiotic stresses. Evidence was based on contrasting lignin levels or differential stress responses of lignin biosynthetic pathways in tolerant and intolerant genotypes. While such studies are informative and quite abundant, their weakness is that unrelated crop genotypes presumably differed in many traits apart from lignification, which may also have influenced their tolerance. This category represents the majority of the studies summarized in Table 2.
- The second one includes studies in which genes affecting lignin biosynthesis were specifically manipulated to obtain near-isogenic lines of crops differing in lignin content. This approach was taken in a number of experiments using diverse crop species such as wheat [96], rice [99], tobacco [103, 104, 121, 130], cotton [108], potato [111], and carrot [113]. Either gain of function (to increase lignin) or loss of function (to decrease lignin) mutants were used in these experiments to obtain direct evidence of the involvement of lignin in tolerance against biotic stresses.
- The third category includes studies applying pretreatment with elicitors to induce systemically acquired resistance in crops, which involved enhanced lignin content in pretreated plants. These studies used either pretreatment with biological elicitors such as *Pseudomonas* ssp. [120], fungal extracts [115, 117], chemical compounds such as salicylic acid, dichloroisonicotinic acid, or bion (benzo(1,2,3)thiadiazole-7-carbothioic acid S-methyl ester [109, 119], or mechanical injury [93]. These approaches were shown to enhance lignin levels and, consequently, resistance to biotic stresses in orange fruits [93], cotton roots [109], tomato [115], peanut [117], cucumber [119], and rice [120].

Despite the abundant evidence for the protective role of lignin against biotic stresses, there are also reports in which the lignin level did not positively affect crops resistance. Lignin concentration was not a factor differentiating *Fraxinus* cultivars resistant or susceptible to emerald ash borer [182]. Also, lignin levels did not explain the resistance of five different switchgrass (*Panicum virgatum*) populations to aphids and aphid-transmitted virus diseases [183]. In sorghum, low lignin “brown-midrib” genotypes even exhibited reduced colonization by *Fusarium* ssp. and *Alternaria alternata *[184]. The authors suggested that impairment of lignin synthesis could shift intermediates of the phenylpropanoid pathway to different branches, which also have protective effects against pathogens. Overall, these exceptions are relatively rare and do not necessarily contradict the majority of studies supporting the important role of lignin in plant defense against biotic stresses. 

Because the cell wall constitutes such an important physical barrier, pathogens have evolved a broad array of enzymes to digest lignocelluloses [185]. A class of fungi called white rot fungi possesses the particular ability to decompose lignin via extracellular enzymes such as lignin peroxidase, manganese peroxidase, and laccase [186–188]. These fungi are increasingly being used in agricultural or industrial applications that require the removal of lignin from plant material, such as ruminant nutrition or bioethanol production [189–191]. The digestive systems of herbivorous insects do not have the capacity of decomposing lignin, but some insects host lignin degrading fungi in their stomachs to facilitate the digestion of lignified plant material [192]. 

## 6. Role of Lignin in Animal Nutrition 

From a point of view of animal nutritionists, lignin represents an undesired or “antinutritive” component. Being part of the cell wall, lignin forms a limiting factor especially in the diets of ruminant herbivores, which unlike monogastric animals are able to digest cell wall material efficiently. With the aid of their anaerobic rumen microbial population, ruminant herbivores ferment polysaccharide polymers (cellulose and hemicelluloses) into short chain fatty acids, which serve as a source of energy for the animal, while the microbes themselves form a source of protein [193, 194]. The extremely diverse rumen microbial population produces many glycosyl hydrolases, that is, enzymes that hydrolyze the glycosidic bonds between carbohydrates, or between carbohydrate and noncarbohydrate molecules [193]. In contrast, lignin is not readily fermented by the rumen bacteria but is only partly degraded by rumen anaerobic fungi [193]. As a consequence, it limits the feed value of plant materials through two mechanisms. (i) Inaccessible energy content: although lignin has about 30 percent higher gross energy content than cellulose [195], this energy is barely accessible for ruminants. Therefore, the lignin content is negatively correlated with digestible energy in ruminant diets. (ii) Reduced feed intake: due to the association with polysaccharide constituents, lignin forms a physical barrier and thus hinders the access of rumen microbes to fermentable cell wall components. Consequently the passage rate of feeds through the rumen is slowed down, thus reducing the feed intake capacity [196, 197]. 

The negative correlation between lignin content and digestibility of forage materials in ruminant diets has been documented in numerous experiments. A common approach to determine the feed value of plant materials for ruminates involves the incubation of samples in rumen liquor *in vitro* to measure digestibility. This can be accompanied by time-course measurements of the amount of gas produced during fermentation, which is positively correlated with digestibility [198]. Using such techniques, lignin was identified as a dominant factor limiting the feed value in perennial grasses [199], maize stems [200, 201], and tropical forages [202]. In an experiment with alfalfa, it was shown that lignin content had a more negative effect in long-term than in short-term *in vitro* incubations, indicating that it affected the potential extent of digestion rather than the rate of digestion [203]. Similar relationships were also found in artificially modeled diets: in incubation experiments with maize cell walls that were artificially lignified using monolignol treatments, the lignification caused up to a 12-fold increase in the lag time of cellulose fermentation [204]. Animal feeding experiments in principle confirmed these *in vitro* experiments. For example, it was demonstrated that lignin was the main chemical parameter explaining the *in vivo* organic matter digestibility of 64 different grass silages fed to cattle [205]. Another study reported a negative correlation between lignin concentration and *in vitro* digestibility in 36 different forages including legumes, C3 grasses, and C4 grasses and confirmed these results in feeding trials with lambs [206]. 

Models have been established to predict cell wall digestibility from the degree of lignification. Traxler et al. [207] identified highly positive correlations between lignin content and the indigestible cell wall fraction of 145 different forages and used these data to develop models for predicting digestibility based on lignin concentrations. Similarly, Kramer et al. [22] concluded that the indigestible fraction of plant materials can be estimated from the lignin concentration but also recommended that the same models cannot be applied across different species. 

In contrast to the well-documented negative impacts of lignification on forage digestibility, a few studies have also reported positive effects of lignin in ruminant diets relating to greenhouse gas emissions. Ruminant production is one of the most important sources of anthropogenic methane, which constitutes the second most important greenhouse gas next to carbon dioxide [208]. In the rumen, methane is produced during the anaerobic fermentation of organic materials, and released into the atmosphere [209]. When purified lignin was added to lamb diets at different rates, it reduced the feed intake but did not affect growth performance. However, it decreased the methane release in *in vitro* incubations of lamb feed formulations [210]. Similarly, high lignin diets exhibited relatively low methane release during *in vitro* incubations in rumen liquor obtained from a cow compared to high sugar diets [211]. In addition, when different types of roughage were incubated in buffalo inoculum, a negative correlation between lignin content and methane release was noted [212]. Together, these studies suggest a positive role of lignin in mitigating methane emissions from ruminant production. 

Despite these rare examples of positive effects of lignin, animal nutritionists usually seek to minimize the lignin content of ruminant diets. Two strategies are discussed in the scientific literature: (i) pretreatment of forages to remove lignin prior to feeding them to animals and (ii) breeding of novel low lignin genotypes of forage crops. 

Pretreatments to limit the negative effect of lignin on forage digestibility include biological, physical, and chemical processes [213]. The most widely used biological pretreatment of forages involves the use of white rot or brown rot fungi. These lignin degrading fungi produce several types of extracellular oxidative enzymes such as laccases and lignolytic peroxidases [214]. This ability has been used to improve the feed value of low quality forages such as wheat straw [215–217], rice straw [218], oil palm fronts [219], Bermuda grass [220], and bamboo [221], just to name a few. In these studies, plant materials were incubated either with fungal inoculum or with isolated enzymes [217] for several days up to fifteen weeks. These treatments were shown to effectively decrease lignification of forages and thus improve their digestibility in ruminant diets. Physical pretreatments such as grinding and steaming usually aim at improving the access of rumen microbes to fermentable cell wall components [213] but may not directly affect lignin content [222]. Chemical treatments involve the extraction of lignin from forages using solvents such as NaOH/ethanol [223] or oxidants such as peracetic acid or hydrogen peroxide [213]. 

Efforts to breed for low lignin content in forage crops comprised both conventional breeding and biotechnological approaches [224]. Selection for high *in vitro* digestibility in four perennial forages was associated with simultaneous selection for low lignin content [47]. Naturally occurring or induced brown midrib mutations are known to reduce the lignin content in a number of grass species [174] and were also associated with improved digestibility in maize [200, 225, 226], sorghum, and sudangrass [227, 228].

A number of studies also tested crop or model species that were genetically modified to contain lower lignin content. The downregulation of different monolignol biosynthetic genes to engineer transgenic alfalfa plants containing less lignin led to improved digestibility in independent experiments [229, 230]. Similar results were reported from transgenic maize in which a gene encoding a lignin biosynthetic gene was suppressed [231]. In contrast, changes in lignin composition (but not lignin quantity) due to manipulation of a gene involved in monolignol synthesis (ferulate-5-hydroxylase) did not affect the *in vitro* digestibility of *Arabidopsis thaliana* plants [232]. Together, these studies clearly demonstrate that genetic approaches are effective in reducing the lignin content and improving the digestibility of forages. 

## 7. Role of Lignin in the Bioenergy Sector

Lignocellulosic crops or crop residues constitute one of the most abundant resources for the expansion of the renewable energy sector [9, 233, 234]. The role of lignin for energy production from biomass is ambivalent. Whether it constitutes a desired or undesired component essentially depends on the energy conversion process. In thermochemical conversion processes, especially in direct combustion, high lignin content improves the energetic value of biomass. It contains less oxygen than cellulose and hemicellulose and has a heating value of 22–24 kJ g^−1^, which is 30 to 50 percent more than that of other cell wall components such as cellulose and hemicellulose [195, 235, 236]. In contrast, lignin is inhibitory to biological conversion processes such as microbial fermentation for bioethanol or biogas generation [235, 237]. 

In direct combustion of lignocellulosic material, the heating value of biomass is strictly positively correlated with its lignin content [238, 239]. Direct combustion has several advantages in small-scale applications: it is straightforward, does not require any processing or investments, and is also cheap and flexible [238]. These potential advantages favor the use of crop byproducts as a source of energy in homes and small industries in developing countries [238, 240]. However, direct combustion has the disadvantage of substantial air pollution and low energy density of unprocessed biomass, making large scale storage and transport unprofitable [238]. Therefore, lignocellulosic biomass is usually processed into more practicable forms such as liquid fuels or combustible gases. 

Cell wall material, including cellulose, hemicelluloses, and lignin, can be converted to liquid fuels by pyrolysis. This thermochemical process involves high-temperature heating in the absence of air or oxygen to produce a pyrolysis oil, a complex mixture of components that is generally a low-quality fuel in itself, but can also be upgraded by further processing [241]. Pyrolysis oils are very diverse in their composition, as illustrated by a study which identified 167 different compounds in the pyrolysis oil obtained from rice husks [242]. Pyrolysis has the added benefit of producing char as a byproduct, a stable carbon sink which can be used as a natural soil amendment (so-called “bio-char”), and sequester carbon dioxide [243]. Due to its chemical structure and highly cross-linked nature, lignin has a higher thermal resistance than cellulose and therefore requires higher temperatures for pyrolysis [244]. Boateng et al. [245] compared the performance of 20 alfalfa samples differing in lignin content in two energy conversion processes: high energy pyrolysis and biochemical fermentation by rumen microbes. While biochemical conversion was negatively correlated with lignin content, no negative impact of lignin on pyrolysis yield was noted. Similarly, Fahmi et al. [246] suggested that lignin did not negatively affect pyrolysis yield, but it may lead to the presence of unstable high molecular weight compounds in the pyrolysis oil, which lower the oil quality. Hodgson et al. [247] found little variation in lignin-derived pyrolysis products in a set of *Miscanthus* genotypes differing in their lignin content and concluded that a substantial proportion of the lignin remained unpyrolyzed at the temperature used in their study (500°C). Another study demonstrated that corn stover pretreated with white rot fungi to break down lignin polymers prior to pyrolysis improved the efficiency of thermochemical conversion of lignin [248]. These examples illustrate that the efficiency and quality of lignin pyrolysis products are variable due to the complex structure and heterogenic composition of lignin. Pyrolysis of lignocelluloses therefore requires optimization of the processing conditions based on the particular species and applications in mind. 

Gasification constitutes an alternative thermochemical conversion process. It involves the conversion of solid biomass to syngas (CO + H_2_) at high temperatures (usually >700°C) with controlled amounts of oxygen, steam, or a mix of gases [249]. After some purification steps, syngas is used in gas turbines or catalytically converted to liquid fuels such as ethanol, although this process remains technically challenging [250]. The gasification of lignin produces four times more hydrogen than cellulose and almost four times more than hemicelluloses [251]. Therefore, high lignin content is considered a favorable trait in biomass used for gasification, and pretreatments often aim at increasing the lignin content. Composting was shown to effectively increase the lignin content of different types of biomass (*Leucaena leucocephala, Chamaecytisus palmensis*), which in turn led to increases in hydrogen yield in gasification [252, 253]. In summary, it can be concluded that direct combustion and gasification constitute the most effective thermochemical conversions processes for high lignin biomass [236], while pyrolysis may lead to variable results. 

In biological energy conversion processes, lignin poses problems very similar to those experienced by animal nutritionists as it constitutes an indigestible component and a mechanical barrier to microbial fermentation of cell wall polysaccharides. The production of bioethanol involves saccharification of cell wall carbohydrates, that is, the enzymatic hydrolysis of cell wall polysaccharides into simple sugars by inoculation with cellulases, followed by fermentation into ethanol by yeast species such as *Saccharomyces cerevisiae *[254]. Due to its inhibitory role, lignin is sometimes removed from biomass prior to saccharification using biological or chemical pretreatments [254]. 

The inhibitory role of lignin in bioethanol production has been demonstrated in many studies. For example, lignin content was negatively correlated with sugar release during saccharification of wheat straw [255]. A similar relationship was reported for the bioenergy species *Miscanthus*, where lignin content was the major determinant of enzymatic biomass degradation [256]. Some authors undertook simultaneous measurements of feed value of forages in ruminant diets and potential ethanol yield and found positive correlations as expected. For example, corn stover *in vitro* digestibility was positively and lignin content was negatively correlated with ethanol yield in corn stover [257]. Anderson et al. demonstrated a positive correlation between ethanol yield and digestibility for ruminants in 50 Bermuda grass accessions, but lignin explained only a small proportion of the variation [258]. Similar results were obtained in experiments with transgenic crops in which lignin level had been manipulated. Transgenic alfalfa plants engineered to contain less lignin than their wild type showed improved saccharification efficiency [259]. Similarly, the reduction of the lignin content in switchgrass by downregulation of a lignin biosynthesis gene improved the ethanol yield by up to 38% and reduced the need for pretreatment of feedstock, as well as the doses of cellulases required for saccharification [260]. While lignin clearly limits the ethanol production from biomass, the unfermented residues of saccharification and fermentation, which contain high levels of lignin, can be reused for thermochemical energy conversion, especially direct combustion, to produce heat and electricity [261]. Alternatively, lignin may be removed from the biomass prior to the saccharification and recovered for diverse applications using chemical precipitation methods [262]. 

An alternative biological method to process biomass into energy is the generations of biogas using anaerobic microbial digestion. The term biogas refers to a mix of combustible gases such as methane and hydrogen, which are formed by mixed microbial cultures digesting biomass in anaerobic reactors [263, 264]. There is broad agreement in the scientific literature that lignin is a major factor limiting the biogas yield in anaerobic digestion [264], as illustrated by both experimental and modeling studies. For example, the methane yield in a variety of crops (maize, sorghum, and *Miscanthus*) depended mostly on the polysaccharide to lignin ratio of the feedstock [265]. Another study tested the biogas production of 57 different plant samples and concluded that a lignin (ADL) content of 10% was a critical threshold for high biodegradability in anaerobic digestion [237]. Also, a significant negative correlation between lignin content and methane production was observed in 285 different maize genotypes [266]. Several predictive models to estimate the biogas yield from lignocellulosic material include lignin as the major negative factor [267–269]. Because lignin is such an important factor limiting biogas yield, pretreatments of feedstock often aim at removing lignin from biomass [270, 271]. Such pretreatments include the chemical extraction of lignin using a variety of solvents [270], biological treatments with lignin degrading fungi or enzymes [270, 272, 273], oxidation of lignin using oxidants such as hydrogen peroxide [274], or heat treatments in combination with extraction or oxidation [275]. 

Regarding the role of lignin, the challenges faced in animal nutrition and in the bioenergy generation via biological conversion are very similar and require better understanding of lignin synthesis and its breakdown. A common strategy that has been proposed in both fields of research is the engineering of modified lignin polymers that are less inhibitory to enzymatic breakdown, while maintaining the functional roles of lignin in adaptation of crops to abiotic and biotic environmental conditions [276, 277].

## 8. Role of Lignin in Soils 

In agricultural soils, lignin has important implications for the soil organic matter (SOM) cycling, thus affecting soil structure, mineralization of nutrients, and carbon (C) sequestration. Traditionally it was assumed that lignin forms a relatively stable component of SOM due to its recalcitrant chemical structure and its resistance to microbial degradation. In this model, the predominant fate of lignin derived from crop residues is the conversion into relatively stable humic substances *via *aromatic residues of lignin polymers [278, 279]. However, this concept is under debate since more recent research suggests that selective preservation of lignin occurs only in the earlier stages of litter decomposition, whereas found that lignin derived compounds do not accumulate in the refractory C pool of soils [280–284]. The low level of associations of lignin with soil minerals has been put forward as a possible explanation for its low accumulation in stable carbon pools [284, 285]. Breakdown of lignin in soil is a predominantly aerobic process mediated by microorganisms such as basidiomycete fungi (brown rot and white rot fungi) and a few species of bacteria such as *Streptomyces* spp. [278, 286, 287]. These microorganisms produce extracellular enzymes such as phenol oxidases and POX [288] that are also employed in pretreatments of ruminant feed or bioenergy feedstock as described in the previous sections. Besides this predominant biotic decomposition of organic matter, abiotic decomposition also occurs due to photo-degradation. Lignin was shown to be more susceptible to photo-degradation than other SOM components because it acts as an effective light absorbing compound over a wide range of wavelengths [289]. Soil scientists have developed indicators to characterize the lignin degradation state in soils, such as the acid (Ac)/aldehyde Ac/Ald ratio, which is determined after the oxidation of samples with CuO to release single ring phenolics. Ac/Ald indicates the ratio of oxidized (carboxylic acid) to more reduced (aldehyde) forms of lignin derived phenolics (such as vanillic acid to vanillin) and increases upon biodegradation of lignin [286, 290]. 

Despite the controversy regarding the long-term fate of lignin in soils, there is broad agreement that lignin is a factor that slows down the mineralization of nutrients from crop residues on the time scale of a cropping season. The lignin concentration and the lignin/N ratio are widely used as indicators for the degradability of litter [291, 292]. For example, lignin negatively affected the short-term N release in rice soils from different types of green manure differing in lignin content, including legumes, azolla, and rice straw [293, 294]. Similarly, N release rate was limited by the lignin/N ratio in a study testing mineralization of nutrients from 12 different plant materials in tropical hillside soils [295]. Remarkably, the authors of this study suggested that nutrient mineralization rate from green manures can be estimated by feed value analyses such as *in vitro* dry matter digestibility, which was confirmed in an investigation of a range of subarctic plant species [296]. The influence of lignin on organic matter decomposition is time dependent and becomes more dominant as decay proceeds, as illustrated in several studies. Taylor et al. [292] suggested that lignin/N ratio was a poor indicator for litter decomposition during the first two months of organic matter incubation in soil but it became more dominant thereafter. This result is congruent with another study [297], in which N released from tropical manure incorporated into soil was not correlated with lignin levels during the first eight weeks of incubation. Similarly, the dissolved organic carbon released from litter decomposition was not affected by lignin during the first five months of litter decomposition, but thereafter it was affected by lignin quantity and quality [298]. In a study on maize roots from 16 different genotypes differing in lignin content and composition, lignin showed no correlation with cumulative C mineralization during the first two weeks of incubation in soil, but showed significant negative correlation from two weeks up to 26 months of incubation [299]. Differences in time scales between studies may be explained with different types of organic matter, different soils, and incubation conditions used. Lastly, lignified biomass with slow mineralization of nutrients could also be interpreted positively as a sustainable fertilizer. Congruent with this concept, artificial ammonoxidized lignin was suggested as a soil amendment combining slow but sustainable nitrogen release with a carbon sequestration function [300]. 

SOM contains two-thirds of the terrestrial C storage in the world [290, 301] and therefore forms a crucial C sink with respect to global change. Lignin is considered to play an important role in C sequestration in soils [302] and is typically considered a recalcitrant carbon pool in models estimating the CO_2_ release from SOM decomposition [291]. In addition, a number of experimental studies proposed beneficial roles of lignin in C stabilization in soils. Dijkstra et al. [303] monitored SOM decomposition as affected by N inputs, plant species, and elevated CO_2_ and concluded that the lignin content of plant litter was a crucial factor determining C stabilization in a grassland ecosystem. Similarly, high lignin content of soil amendments such as compost was considered as a factor leading to stabilization of soil organic C in nonlabile pools in flooded rice ecosystems [304]. In contrast, an increasing number of studies found that lignin derived compounds did not selectively accumulate in the refractory C pool of deeper soil horizons [280–283], suggesting that the role of lignin in long-term C sequestration remains to be fully elucidated. Moreover, feedback reactions of global change on carbon cycling in soils are expected, because processes such as lignin decomposition are influenced by environmental factors such as rising temperatures [305]. In a soil warming experiment, the presence of lignin degrading fungi was increased and the degradation of lignin was accelerated at higher temperatures, which would imply faster rates of lignin decomposition in future climates [306]. In contrast, when soil samples were taken from 18 different grassland sites across temperature transects, cool climate favored higher Ac/Ald ratio, indicating a higher degree of microbial lignin decomposition [285]. Such apparent contradictions might be explained with different methodological approaches, that is, a single site experiment in which one factor (soil temperature) was varied versus a multisite study. In conclusion, important questions regarding the potential of lignin to contribute to carbon sequestration in a changing environment are under debate and remain to be answered. 

## 9. Lignin as a Target for Crop Breeding

Lignin content or composition is not a classical target trait in crop breeding, which tends to be more focused on crop yields, stress resistance, or consumer quality of edible crop parts. More recently, molecular breeding techniques in combination with high throughput phenotyping have allowed for more targeted inclusion of specific quality traits such as lignin content in crop breeding schemes. As detailed in the previous sections, lignin content of crops potentially constitutes an important breeding target from the perspectives of several agricultural disciplines. In the past, crop breeding projects were motivated mostly by the role of lignin in animal nutrition and biofuel production and thus aimed at decreasing the lignin content of crops. In principle, three approaches have been adopted in breeding crops with modified lignin content: (i) use of naturally occurring or induced brown midrib mutations, which affect the lignin content; (ii) mapping of quantitative trait loci (QTL) influencing the lignin content, which can be applied in marker assisted selection; (iii) genetic modification of lignin biosynthetic genes to generate transgenic crops with altered lignin content or composition. 

Brown midrib mutations were discovered in the 1920s in maize plants, which showed reddish-brown pigmentation of the leaf midrib [307]. Subsequently, four genes causing this phenotype in maize were identified that originated from natural mutations and were named *bm1*, *bm2*, *bm3,* and *bm4*. It was later discovered that these mutations caused reduced lignin levels [307]. Meanwhile, brown midrib mutants have been isolated in other C4 grasses such as sorghum and pearl millet, arising either through spontaneous or chemically induced mutations. Some genes underlying brown midrib loci have been identified in maize and sorghum. They encode orthologues of lignin biosynthetic genes such as caffeic-O-methyltransferases and O-methyltransferase [174, 308]. Moreover, candidate genes for a further brown midrib locus in maize (bm6) were proposed by genetic mapping [309]. Brown midrib mutants form an excellent model for investigating implications of lignin for crops and have been characterized regarding their resistance to biotic stresses [184], feed quality [227, 310, 311], biofuel potential [312–314], and degradability in soil [315]. 

QTL mapping and marker assisted selection take advantage of the naturally occurring genetic variation in lignin content occurring within crop species [20, 266, 316, 317]. QTL associated with lignin content were reported for a number of crop species (Table 3). The majority of these studies dealt with maize, but a few studies also investigated other species such as barley, sorghum, and rice (Table 3). The primary research objective of most experiments was to improve the feed quality for ruminants by lowering the lignin content, and lignin content was only one among several feed quality parameters for which QTL were reported. However, a few studies, especially on sorghum, were primarily designed to increase the bioenergy potential of the crop (Table 3). In most populations, a fairly large number of QTL were detected with low or intermediate effects, individually explaining up to twenty percent of the phenotypic variation in lignin content, as indicated by partial *R* ^2^ values (Table 3). In maize, only one major QTL explaining 43 percent of Klason lignin (KL) content was reported in a recent study [142]. Remarkably, this latter study found no colocalization between QTL for KL and ADL, indicating that these two types of lignin represent different fractions of the cell wall. Summarizing their work with six different mapping populations of maize, Barrière et al. [142] assembled an inventory of 50 QTL for ADL, which corresponded to 23 positions in the maize genome. Some QTL for lignin content in crops colocalized with *in vitro* dry matter digestibility [316], genes involved in lignin biosynthesis [135], or regulatory elements involved in cell wall synthesis [142]. Surprisingly, in some maize populations (e.g., [138]) no colocalization of QTL for lignin and digestibility was observed, indicating that other factors were limiting the digestibility for ruminates. Apart from maize, a major QTL was reported in rapeseed explaining 39 percent of the variation in ADL [149]. Subsequent fine mapping and sequencing of a candidate gene revealed that a polymorphism in the lignin biosynthetic gene cinnamoyl Co-A reductase 1 was probably responsible for differences in seed lignin content. Besides classical QTL mapping for simple quantitative traits such as lignin content, eQTL (expression quantitative trait locus) mapping has more recently been developed to identify genomic regions associated with gene expression patterns associated with a particular phenotype. This approach was used by Shi et al. [318], who selected 439 candidate genes associated with altered cell wall composition in brown midrib maize mutants, and determined eQTL regulating their expression. 

While QTL experiments, such as those summarized in Table 3, typically use biparental populations, genome-wide association mapping is emerging as a powerful tool to map genes for quantitative traits in populations of unrelated individuals. This approach has the advantage of sampling more genetic diversity and avoids time consuming generation of crosses necessary for QTL mapping [319]. A genome-wide association study identified loci associated with leaf metabolites in 289 diverse maize lines genotyped with 56 110 SNP markers and reported a locus significantly associated with the level of the lignin precursor *p*-coumaric acid, which was also correlated with lignin content [320]. 

Alternatively, lignin content of crops was modified by transgenic approaches [321]. Genetic engineering strategies included the manipulation of lignin biosynthesis at the regulatory level, controlling monolignol biosynthetic enzymes, and modification of lignin polymer structure [277, 321]. Gene knock-down using RNA interference (RNAi) or antisense techniques was successfully employed in a number of crop species targeting different lignin biosynthetic genes, especially those involved in monolignol synthesis. Silencing of genes encoding cinnamyl alcohol dehydrogenase in maize [322] and alfalfa [229] induced changes in lignin composition rather than notable changes in lignin content but significantly affected the digestibility of transgenic lines. O-methyltransferase genes were down-regulated in transgenic maize [231, 323], alfalfa [324], sugarcane [325], and switchgrass [260]. The suppression of gene expression in these species was associated with a decrease in lignin content by up to 30 percent and altered lignin composition, as well as improved digestibility and bioenergy potential. Similarly, the downregulation of three cytochrome P450 genes involved in monolignol synthesis reduced the lignin content by up to 40 percent and altered the lignin composition in transgenic alfalfa [326]. While all of these studies targeted specific monolignol biosynthetic genes, Fornal et al. [327] identified a transcription factor that suppressed the expression of several monolignol biosynthetic genes and proposed it as a good candidate for manipulating the lignin biosynthesis. In addition to these studies with agricultural crops, a large number of mutants of model plants with altered lignin content or composition have been reviewed previously [328].

Concerns have been raised that the breeding of low lignin crops may unintentionally compromise plant fitness by increasing their susceptibility to abiotic or biotic stresses, or by decreasing the plant rigidity and biomass yield [47, 329, 330]. A possible solution to this dilemma could be the breeding of plants with altered lignin composition, which would be less inhibitory towards microbial fermentation in ruminant diets of bioenergy production. More specifically, the substitution of traditional monolignols by alternative monomers with reduced hydrophobicity or cross-linking to structural carbohydrates has been proposed [276, 331, 332]. 

## 10. Summary and Conclusions

**Figure 3:**
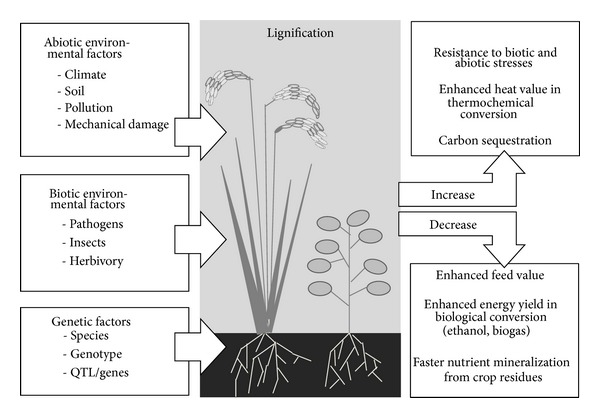
Conceptual model of factors influencing the lignification of crops and its implications for different agricultural applications.

The previous sections elucidated the processes and factors affecting lignin deposition in crops, as well as the sometimes conflicting role of lignin in various agricultural disciplines. To summarize these considerations, a conceptual model of factors determining lignification of crops and implications for the utilization of lignocellulosic biomass is suggested (Figure 3). Lignification depends on many abiotic and biotic environmental factors. In particular, the presence of environmental stresses tends to increase lignification in most cases, as detailed in the respective sections of this review. Moreover, the lignin content of crops depends on genetic factors such as species, genotype, and specific genes or loci, which are exploited in the breeding of crops with altered lignin content or composition. There are also numerous genotype-by-environment interactions influencing lignification, as evidenced, for example, by the fact that many of the QTL studies summarized in Table 3 detected completely different QTL for lignin content when the same populations were grown in different environments. A better understanding of such genotype-by-environment interactions may be one of the major challenges in developing crops with customized lignin content or composition. 

Whether high or low lignin is desired depends largely on the use of lignocellulosic material. Applications that favor high lignin content include the breeding of crops resistant to biotic and abiotic stresses, the use of biomass in thermochemical energy conversion processes, and carbon sequestration in recalcitrant biomass (Figure 3). On the other hand, applications that favor low lignin content include the feeding of biomass to ruminant herbivores, biological energy conversion processes such as ethanol or biogas production, and use of crop residues as a nutrient stock for subsequent crops. Exploiting synergies and harmonizing the apparently conflicting roles of lignin remain a major challenge for research, which requires interdisciplinary approaches. A growing number of studies take account of these diverse perspectives by bridging different disciplines. For example, feed digestibility tests using rumen liquor have been used to estimate the degradability of biomass in other media such as soil [295, 296] or bioenergy reactors [257]. Wang et al. [99] reported that enhanced lignin content due to the overexpression of a transcriptional regulator conferred tolerance to both abiotic stress (UV-B) and biotic stresses such as rice blast and white backed planthopper. Breeders have recognized that the breeding for low lignin content to enhance the biological degradability of lignocelluloses may compromise plant fitness and stress resistance [46], although this problem may be overcome by manipulating lignin composition instead of lignin content [276]. However, harmonizing all of the conflicting roles of lignin in the diverse disciplines may not always be possible, thus necessitating priority setting regarding the use of lignocellulosic biomass.

## Figures and Tables

**Table 1:** Effect of abiotic stresses on lignin concentration of different morphological fractions of crops.

Species/morphological fraction	Effect on lignin	References
Drought		
Forage legumes	↓↑	[48–52]
Forage grasses	↓↑—	[53–55]
Maize leaves, *Zea mays* L.	↓↑—	[56, 57]
Barley straw, *Hordeum vulgare* L.	↑	[58]

Salinity		
Lettuce roots, *Lactuca sativa* L.	↑	[59]
Tomato roots,* Solanum lycopersicon *L.	↑—	[60, 61]
Bean roots*, Phaseolus vulgaris* L.	↑	[62]
Maize roots, *Zea mays* L.	↑	[63]
Rice root, *Oryza sativa* L.	↓↑	[64, 65]
Soybean root, *Glycine max* L.	↑	[66]

Mineral toxicities (Al, B, Cd, Cu, Mn)		
Chamomile root, *Matricaria chamomilla* L.	↓↑—	[67]
Rice roots	↑	[68]
Wheat roots, *Triticum aestivum* L.	↑	[69, 70]
Flax roots*, Linum usitatissimum* L	↑	[71]
Soybean roots	↑	[72, 73]
Tomato roots	↑	[74]

Mineral deficiencies (Ca, K, Mn, N, P, Si)		
Wheat root and shoot	↓	[75, 76]
Chamomile root	↑	[77]
Tobacco root/shoot, *Nicotiana tabacum* L.	↑	[78]
Soybean root	↑	[79]
Potato tubers, *Solanum tuberosum* L.	↑	[80]
Rice shoot	↑	[81]

Ozone		
Rice straw	↑	[32, 34, 82]
Forage legumes	↑	[83, 84]
Forage grasses	↑	[85–87]

UV		
Forage grasses	↑	[88]
Tomato fruit	↑	[89]
Cucumber seedlings, *Cucumis sativus* L.	↑	[90]
Quinoa seedlings; *Chenopodium quinoa* Willd.	↑	[91]
Soybean leaves	↓	[92]

↑ indicates that exposure to stress induced an increase in lignin content, ↓ indicates that exposure to stress induced a decrease in lignin content; — indicates that exposure to stress had no clear effect on lignin content.

**Table 2:** Summary of studies suggesting lignification as an effective defense mechanism against biotic stresses in agricultural crops.

Crop species	Pathogen species	References
Fungi		
Orange fruits*, Citrus sinensis *L.	*Penicillium digitatum *(Pers.: Fr.) Sacc.	[93]
Apple fruit*, Malus domestica *L*. *	*Penicillium expansum *Link	[94]
Einkorn wheat, *Triticum monococcum *L.	*Blumeria graminis*f. sp. tritici (Bgt).	[95, 96]
Wheat, *Triticum aestivum* L.	*Fusarium graminearum *Schwabe Pyrenophora tritici-repentis (Died) Drechsler	[97] [98]
Rice, *Oryza sativa* L.	*Magnaporthe grisea *(T.T. Hebert) M.E. Barr	[99, 100]
Perennial ryegrass, *Lolium perenne *L.	*Puccinia coronata *Corda f.sp. lolii Brown	[101]
Camelina*, Camelina sativa *L. Crantz	*Sclerotinia sclerotiorum *(Lib.) de Bary	[102]
Tobacco, *Nicotiana tabacum* L.	*Botrytis cinerea *(De Bary) Whetzel, *Pythium *ssp., *Alternaria *ssp.	[103, 104]
*Medicago truncatula* Gaertn.	*Phoma medicaginis* Malbr. & Roum.	[105]
Raspberry, *Rubus *ssp.	*Didymella applanata* (Niessl) Sacc.	[106]
Soybean, *Glycine max* L.	*Phakopsora pachyrhizi *Syd. & P. Syd	[107]
Cotton, *Gossypium hirsutum* L.	*Fusarium oxysporum *Schlechtend *Pythium debaryanum *R. Hesse *Verticillium dahliae *Kleb.	[108–110]
Potato, *Solanum tuberosum* L.	*Phytophtora infestans *(Mont.) De Bary *Alternaria solani *Sorauer.	[111, 112]
Carrot, *Daucus carota* L.	*Mycocentrospora acerina *(R. Hartig) Deighton *Alternaria radicina *Meier, Drechsler & E.D. Eddy	[113, 114]
Tomato, *Lycopersicon esculentum* L.	*Fusarium oxysporum *f. sp. lycopersici	[115]
Pearl millet*, Pennisetum glaucum (L.) R.Br *	*Sclerospora graminicola *(Sacc.) J. Schröt.	[116]
Peanut, *Arachis hypogea* L.	*Sclerotium rolfsii *(Curzi) C.C.Tu & Kimbr.	[117]
Pepper, *Capsicum annuum* L.	*Verticillium dahliae *Kleb.	[118]
Cucumber, *Cucumis sativus* L.	*Colletotrichum orbiculare *(Berk. & Mont.)	[119]

Bacteria		
Rice	*Xanthomonas oryzae *	[120]
Tobacco, *Nicotiana tabacum* L.	*Erwinia carotovora *	[121]
Tomato	*Ralstonia solanacearum *	[122]

Nematodes		
Banana, *Musa paradisiaca* L.	*Radopholus similis *(Cobb) Thorne*, Pratylenchus coffeae *Goodey	[123–125]
Tomato	*Meloidogyne incognita *	[126]
Soybean	*Heterodera glycines *Ichinohe	[127]

Insects		
Rice	*Sogatella furcifera *Horváth	[99]
Maize, *Zea mays* L.	*Ostrinia nubilalis *Hübner, *Sesamia nonagrioides *Lefèbre	[128, 129]
Tobacco roots	*Agriotes *spp.	[130]
51 grassland species	Multiple	[131]

**Table 3:** Summary of studies reporting QTL for lignin content of different crop species.

Species	Population	Marker type	Lignin type	No. of QTLs detected (partial *R* ^2^)	Primary breeding aim	Reference
Maize	100 RIL of F2 (—) X Io (—)	152 RFLP	ADL	1 (7.6)	Forage quality	[132]
Maize	131 RIL of F288 X F271	341 SSR	ADL/KL	21 (6.6–20.4)	Forage quality	[133]
Maize	200 RIL of B73 (↓) X B52 (↑)	120 RFLP, 65 SSR	ADL	Sheath 8 (0.2–12.2) Stalks 12 (0.3–10.4)	Forage quality	[134]
Maize	191 RIL of B73 (↓) X De811 (↑)	113 RFLP, 33 SSR	ADL	10 (6–17)	Forage quality	[135]
Maize	200 RIL of B73 (↓) X De811 (↑)	113 RFLP, 33 SSR	ADL	12 (4–17)	Forage quality	[136]
Maize	242 RIL of F838 (↑) X F286 (↓)	249 SSR	KL/ADL	15 (5.9–16.5)	Forage quality	[137]
Maize	140 RIL of Fl1 (↓) X Fl2 (↑)	189 SSR	ADL	4 (10.7–19.7)	Forage quality	[138]
Maize	240 RIL of F838 X F286	101 SSR	KL/ADL	14 (5.6–21.2)	Forage quality	[139]
Maize	223 RIL of B73 (—) X Mo17 (—)	Maize GDB map^§^	KL	4 (5-6)	Biofuel production	[140]
Maize	206 RIL of B73 (—) X Mo17 (—)	IBM2 framework map^#^	NIRS	6 (18.7–28.1)	Biofuel production	[141]
Maize	163 RIL of RIo (↑) X WM13 (↓)	108 SSR	KL/ADL	15 (8.5–43)	Diverse	[142]
Barley	494 RIL of Arta (↓) x *H. spontaneum* 41-1 (↑)	158 RFLP, 30SSR	NIRS	11 (4.2–8.9)	Forage quality	[143]
Barley	72 DH of Steptoe (—) X Morex (—)	327 markers^$^	ADL	4 (8.6–14.2)	Forage quality	[144]
Sorghum	176 RIL of BTx623 (—) X Rio (—)	68 SSR and 222 AFLP	ADL	Stem 5 (n.a.) Leaf 5 (n.a.)	Biofuel production	[145]
Sorghum	188 RIL of SS79 (↓) X M71 (↑)	157 SSR and AFLP	ADL	15 (7.1–18.9)	Biofuel production	[146]
Rice	127 DH of ZYQ8 (↑) X JX17 (↓)	243 RFLP	ADL	1 (23.8)	Forage quality	[147]
Rice	202 BIL of Xieqingzao (↑) X DWR (↓)	149 markers	ADL	5 (4.9–12.6)	Forage quality	[148]
Rape seed	232 RIL of GH06 (↓) X P174 (↑)	RFLP/SSR	ADL	1 (39.3)	Feed value	[149]

RIL: recombinant inbred lines; DH: doubled haploids; BIL: backcross inbred lines; (↓) denotes parent with lower lignin content, (↑) denotes parent with higher lignin content, (—) denotes no consistent difference in lignin content between parents; RFLP: restricted fragment length polymorphism; SSR: simple sequence repeat; AFLP: amplified fragment length polymorphism; ^§^marker data were obtained from http://www.maizegdb.org/; ^#^marker data were obtained from www.maizemap.org; ^$^marker data were obtained from http://barleygenomics.wsu.edu/; ABSL: acetyl bromide soluble lignin; ADL: acid detergent lignin; KL: Klason lignin; NIRS: lignin content was determined by near-infrared spectroscopy; partial *R* ^2^ indicates the proportion of phenotypic variation explained by individual QTL; n.a.: not available.
